# General Movements trajectories and outcome at 12 months in very preterm infants: An analysis of tendencies and pathological persistence

**DOI:** 10.1038/s41598-023-49037-w

**Published:** 2023-12-09

**Authors:** Uta Teschler, Anne-Kathrin Dathe, Katharina Maria Heuser-Spura, Johanna Bialas, Larissa Jane Cordier, Bilge Albayrak, Ursula Felderhoff-Mueser, Britta Maria Huening

**Affiliations:** 1 Department of Paediatrics I, Neonatology, Paediatric Intensive Care and Paediatric Neurology, University Hospital Essen, University of Duisburg-Essen, Essen, Germany; 2 Center for Translational Neuro- and Behavioral Sciences, University Hospital Essen, University of Duisburg-Essen, Essen, Germany; 3 Department of Health and Nursing, Occupational Therapy, Ernst-Abbe-University of Applied Sciences, Jena, Germany

**Keywords:** Medical research, Outcomes research, Paediatric research

## Abstract

Very preterm infants (VPI) < 32 weeks are at increased risk of developmental disorders detectable using the Prechtl General Movements Assessment (GMA) and the Bayley Scales of Infant and Toddler Development-Third Edition (BSID-III). The aim of this study was to investigate General Movements (GMs) trajectories from preterm to fidgety age including GMs tendencies and their association with cognitive and motor outcome. Retrospective analysis of VPI with GMA at preterm (35 ± 2 weeks postmenstrual age (PMA), T1) and fidgety age (12 ± 3 weeks corrected age CA), T2), and BSID-III (12 ± 3 months CA, T3) is performed. Data are analysed using Pearson χ^2^-test, Fisher-Freeman-Halton Exact test, and residual analyses. This study found significant associations between (a) GMs (T1) and (b) persistent pathological GMs (T1 + T2) with cognitive outcomes at 12 months (T3) considering the tendencies of GMs in addition to the global character (p = 0.007, p = 0.022, respectively), representing medium-sized effects. There were no significant associations between GMs or persistence of pathological GMs and gross and fine motor outcomes, regardless of GMs tendencies. Findings indicate that considering tendencies of GMs and the persistence of pathological GMs may be important in identifying children at risk of cognitive impairments early. This additional assessment parameter may have the potential for early identification of infants with milder motor and/or cognitive impairments. However, more research is needed using larger sample cohorts to generalise the results and to be able to recommend sequential GMA for clinical routine.

## Introduction

Very preterm infants (VPI) with gestational age < 32 weeks postmenstrual age (PMA) and/or birth weight < 1500 g are at increased risk of cerebral injury and alterations in brain development^1–3^. These can significantly affect further development. The spectrum ranges from mild functional impairment to profound disorders. In the EPIPAGE-II study, Twilhaar et al. show the heterogeneity of outcomes in VPI at the age of 5 years: 45% of infants have no impairment. Regarding disabilities the majority has motor and cognitive impairment (31%). In addition, there are psychosocial deficits and conspicuous behaviour (16%), and a small proportion develop multiple deficits in multiple domains (8%)^4^. Cognitive deficits can have an impact on academic success as well as career choice and employment^2,5^. In terms of motor skills, the most severe neurological complication is spastic infantile cerebral palsy (CP)^6^, which currently affects around 6.8% of VPI (pooled prevalence)^7^. Much more common than CP are milder motor impairments, which are described as developmental coordination disorders (prevalence up to 40% in VPI) and which are disproportionately more difficult to predict^8–12^. For early prediction of CP, Prechtl’s General Movements Assessment (GMA) at 3–5 months corrected age (CA) is most suitable due to its high specificity and sensitivity^13,14^. In addition, associations of General Movements (GMs) with motor and cognitive dysfunction have been identified^8,15,16^. The GMA is a non-invasive tool for assessing spontaneous movements of preterm and newborn infants. The movement variability is described in different characters depending on the infant´s age. During preterm and term age, a distinction is made between the global characters: Normal (N), Poor Repertoire (PR), Cramped Synchronised (CS) and Chaotic (CH)^17^. GMs are classified as N if an infant presents involuntary flowing movements with variable speed and amplitude throughout the body. Lack of variability and monotony characterise PR. The stiff-rigid CS with synchronised contractions and relaxation of the extremities and trunk is severely pathologic. CH is characterised by rapid movements with large amplitude and lack of rotations. This pathology is very rare and occurs in the preterm age^17^. Pathological GMs indicate a movement disorder, especially if the pathology is observed during premature infancy and continues for a prolonged period after term equivalent age^18–21^. By considering the developmental trajectory, GMs may change e.g., PR can evolve into CS or N. These intermittent indications of a different character occur temporarily and are referred to as tendencies in this work. That means that a present movements pattern has a predominant global character but in addition has intermitted improved or poorer parts, e.g., PR with a tendency towards CS expresses a predominant monotony with intermitted synchronised cramped legs movements without sudden release. In contrast, PR tending towards N describes the predominant monotony, which is interrupted by initial harmonious and variably flowing movements. At 3–5 months CA, GMs change into smooth movements with fine amplitude, the Fidgety Movements (FMs). FMs are an essential biomarker for predicting the development of CP (sensitivity 98%, 95% confidence interval [CI] [74–100]; specificity 91%, 95% CI [83–93])^13,14^. Age appropriate FMs are visible as small, elegant movements throughout the body^22^ and are classified as continuous (F++) or intermittent (F+). Pathological FMs are described as AF (abnormal) or F− (absent)^19,22,23^. The absence (F−) or abnormality (AF) at 3–5 months CA^19,22,23^ are clear indications of motor or cognitive dysfunction^23–26^. Kahraman et al. found an association between FMs and the scores of the Bayley Scales of Infant and Toddler Development, Third Edition (BSID-III) at 18 and 24 months CA. Infants with F− scored lowest on the motor section of the BSID-III and infants with AF had the greatest language impairments^27^.

In the last decade some studies have analysed the longitudinal trajectory of GMs from preterm to fidgety age^21,28,29^. Porro et al. examined the relationship of change in the classification of preterm GMs to FMs. The authors concluded that a single analysis in the preterm period, resulting in PR, has a low predictive value for the quality of FMs and they thus favour sequential examinations^29^. Kadam and co-authors continue their evaluations to 12 months CA to predict neurodevelopmental outcome at 9 months CA and motor and cognitive outcome at the 12 months CA. The goal of these early (sequential) assessments is to predict even mild motor and cognitive deficits as early as possible. Achieving reliable early prediction even before an MRI at the calculated delivery date would be desirable. Earlier identification of infants at increased risk for neurodevelopmental deficits offers the potential for prompt targeted therapeutic intervention, ideally during the neonatal inpatient period, with the aim of a seamless continuation of outpatient therapy after hospital discharge. This is particularly true for infants with PR, the milder, not age-appropriate GMs, who demonstrate low variability and monotonous movements, and who continue to show these traits beyond the calculated date. However, PR can develop towards N as well as towards the severe stiff-rigid pathology character of CS. The specification of PR with tendency to CS may increase predictability of FMs and outcome at 12 months. To ensure GMs performance in clinical routine even when resources are limited, the "Detailed Assessment of General Movements During Preterm and Term Age: GMOS"^17^ is not used in this study as the GMOS is time and personnel intensive. Longitudinal analysis of GMs, however, is worthwhile because monotonic GMs with low variability (PR) in preterm and term age are unspecific in predicting outcome. The need for early intervention may be identified in the preterm and term period by detecting a change in global character or tendencies. Our aim was to investigate (1) the developmental trajectories of neonatal GMs from preterm to fidgety age and (2) their relationship with BSID-III scores at 12 months CA. Furthermore, we aimed to assess (3) the inclusion of GMs tendencies and (4) the persistence of pathological GMs in relation to outcome.

## Materials and methods

**Figure 1:**
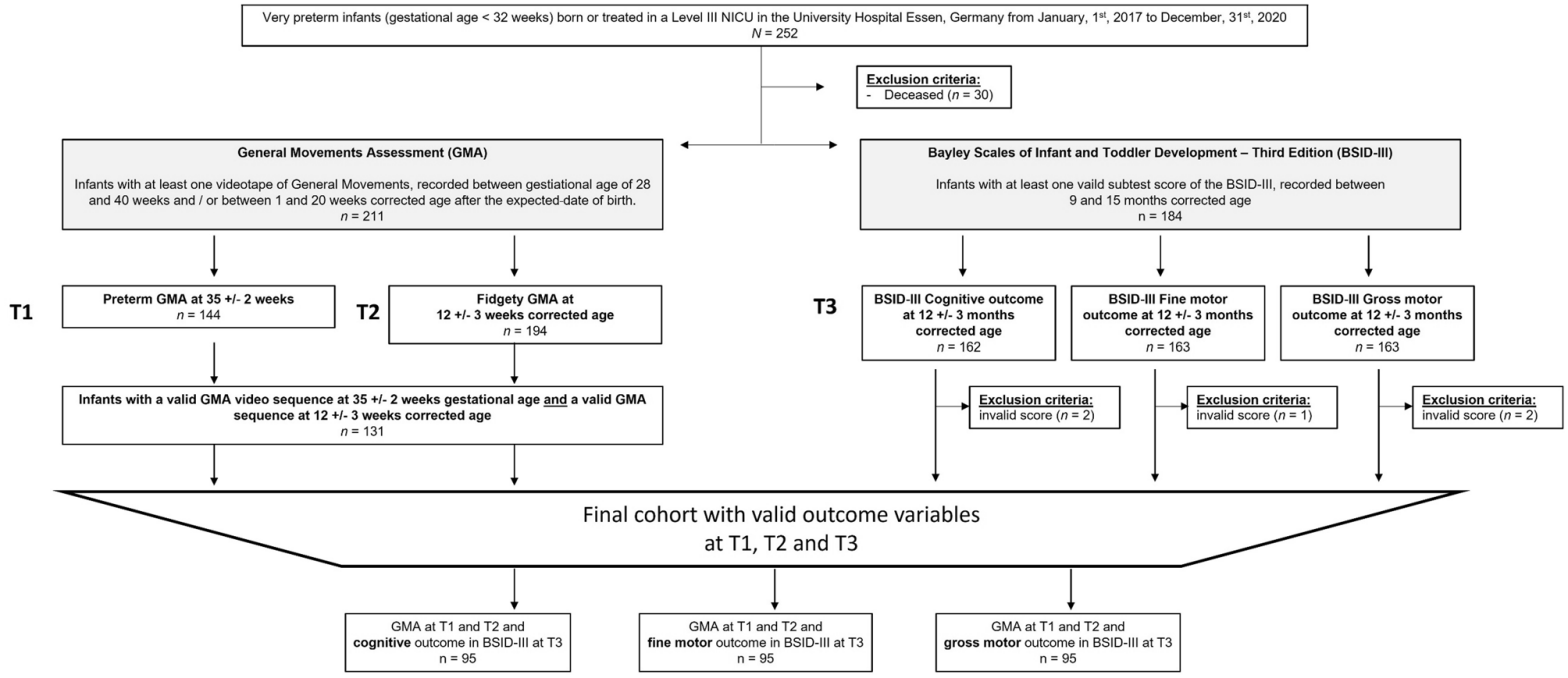
Recruitment cohort over the study period, inclusion and exclusion criteria, and number of infants at outcome time points with the General Movements Assessments (GMA) (35 ± 2 weeks postmenstrual age; 12 ± 3 weeks corrected age) and the Bayley Scales of Infant and Toddler Development Third Edition (BSID-III) (12 ± 3 months corrected age).

This is a retrospective analysis of clinical data of VPI with < 32 + 0 weeks PMA who are treated in a level III neonatal intensive care unit (NICU) between 01.01.2017 and 31.12.2020 (see Fig. 1). The consent of the Ethics Committee of the Medical Faculty of Essen is obtained (ID: 18-8388-BO).

**Figure 2:**
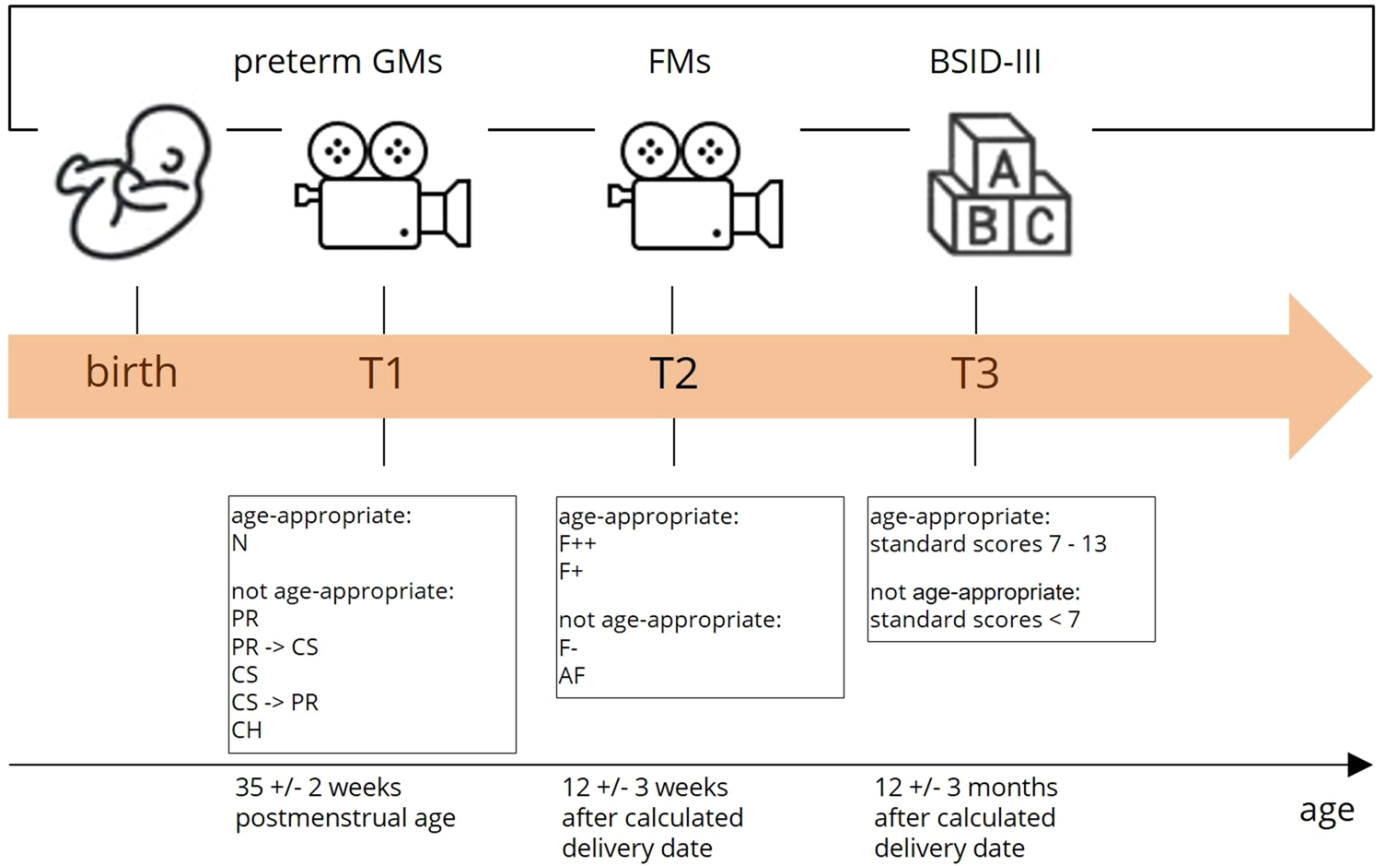
Assessments and possible outcome variables at time points T1, T2 and T3. GMs = General Movements, FMs = Fidgety Movements, BSID-III = Bayley Scales of Infant and Toddler Developments, Third Edition, N = normal, F++ = continuous Fidgety Movements, F+ = intermittent Fidgety Movements, PR = Poor Repertoire, PR -> CS = Poor Repertoire with tendency to Cramped Synchronised, CS = Cramped Synchronised, CS -> PR = Cramped Synchronised with tendency to Poor Repertoire, CH = Chaotic, AF = Abnormal Fidgety Movements.

Three data collection points (T1–T3) are defined for the study. The GMA is performed at T1 (preterm age, 35 ± 2 weeks PMA) and at T2 (fidgety age, 12 ± 3 weeks CA)^19,25^. At T3 (12 ± 3 months CA), the BSID-III is performed (see Fig. 2).

### General Movements Assessment (GMA)

For this study participants are classified with a global character: N, PR, CS or CH GMs, respectively. N is characterised by variable amplitude and speed throughout the body, while PR characterises monotony without variability. CS is stiff and rigid with synchronised contractions of the extremities and trunk followed by sudden relaxation. Chaotic (CH) GMs are fast and huge movements without rotations. GMs can change their quality, e.g., PR can evolve into CS, or it can normalise. Therefore, tendencies are assigned such as an infant may show predominantly PR with short, intermitted moment(s) of synchronised leg or arm extensions that fade in without the abrupt relaxation but instead with a slow decrease in muscle tone (PR with a tendency towards CS, PR -> CS). Another example may be PR with a tendency to N, which means that the monotony is interrupted by brief initiations of harmonious flowing movement in the shoulders and/or pelvis. FMs become visible at 3–5 months CA. Age appropriate FMs are elegant and fluent movements involving the whole body^22^. FMs can be continuous (F++) or intermittent (F+) while pathologies are classified as AF (abnormal) or F− (absent)^19,22,23^. The GMOS^17^ is not used in order to make the GMA more suitable and feasible. Infants are videotaped for 3–5 min using a standardised procedure. Video recordings are quality controlled. Videos are excluded from the study if the infant is crying or screaming persistently, or if there is any interaction/intervention by the caregiver as this prevents appraisal of the GMA. If multiple video files are available at T1 or T2, the video closest to the defined mean of age ranges is used. GMs are scored by at least two advanced certified GMA staff members; if there is disagreement, a third certified staff member scores the movements. The global GMA classifications at T1 are N, PR, CS, or CH and at T2 F+/F++, F− and AF (Fig. 2). PR GMs at preterm age are non-specific in terms of early prediction of FMs outcomes and are not suitable for prognosis^29^. Based on the observable possible changes in the quality of GMs, PR without a tendency to N and PR with a tendency to N are classified as not clearly pathological in the context of this study. CS and PR -> CS or CS with a tendency towards PR (CS -> PR) are attributed as definite pathological findings for data analysis.

### Bayley Scales of Infant and Toddler Development (BSID-III)

The BSID-III provides objective and standardised information on the developmental status of infants aged 1–42 months for the dimensions of cognition, gross and fine motor skills, language, and social-emotional and adaptive behaviour^30^. Scores 1–2 standard deviations (SD) below the normative mean indicate retardation or developmental delay in motor and/or cognitive performance relative to peers^31,32^. The German edition of BSID-III^33^ is administered by qualified and certified staff members with several years of practical experience in BSID-III testing. Reliability is not calculated as the BSID-III scores are obtained from clinical routine. The assessment is done in the presence of at least one parent at the infants' CA of 12 ± 3 months (T3). Staff members are blinded towards the GMs findings. It takes approximately 50–60 min to complete. The subscales of cognitive, gross motor and fine motor scales are defined as outcome variables. Results are considered age-appropriate if the test score is between 7 and 13. Due to the aim of the study, as well as the multicultural composition of the patient population, the language scale is not part of the analysis. Standardised developmental testing is limited from March 2020 to April 2021 due to the SARS-CoV-2 pandemic. Compensatory BSID-III testing via video consultation is unfortunately standardised not possible. The pandemic also has an impact on GMs videos, as home videos do not always meet the required quality standards.

### Data analysis

Data analysis is performed with SPSS 29.0 [IBM SPSS Statistics for Windows, IBM Corp.]. First, the relationship between the global character of preterm GMs (N vs. PR vs. CS) at T1 and FMs (F−, AF, F+, F++) at T2 is tested on the whole sample of N = 131 using Pearson χ^2^ tests for independence. Analyses are repeated taking into account the global character of the GMs in combination with the tendency at T1. Fisher–Freeman–Halton exact tests are reported when more than 20% of cells has an expected frequency below 5. The alpha level is set at 0.05 and is two-tailed for all analyses. To further explore the properties of a significant association, residual analyses are performed based on adjusted standardised residuals and adjusted for multiple testing using Bonferroni correction to avoid inflating type I errors. Effect sizes are reported as Cramer's V and interpreted as 0.10 = small, 0.30 = medium and 0.50 = large effects. Secondary analyses are performed to test the relationship between preterm GMs and BSID-III outcomes and FMs and BSID-III outcomes separately. Therefore, Pearson χ^2^ tests are performed with the GMs global character at T1 and BSID-III outcome variables at T3, as well as FMs at T2 and BSID-III outcome variables at T3, separately for cognitive, fine motor and gross motor subtest scores. The analysis is repeated considering the global character of the GMs in combination with the tendency at T1. Finally, to test the relationship between the persistence of pathological GMs at T1 and T2 and the BSID-III outcome variables at T3, the preterm quality variables for GMs and FMs are recoded. The following categories are obtained: no pathological GMs at both measurement time (i.e., no CS at T1 and no F− at T2), pathological GMs at one measurement (CS at T1 or F− at T2), and persistent pathological GMs at both measurement time (CS at T1 and F− at T2). Pearson χ^2^ tests are performed between the categorical variable persistence of pathological GMs and the binary BSID-III outcome variables (that is, age-appropriate vs. not age-appropriate) separately for cognitive, fine motor and gross motor subtest scores, based on a reduced sample of n = 95. Analyses are repeated taking into account the global character of the GMs in combination with the tendency at T1. Therefore, a second variable of persistence of pathological GMs is calculated with the following categories: no pathological GMs and tendencies at both measurement times (no CS at T1 or no tendency to CS at T1 and no F− at T2), no persistent pathological GMs or tendencies at one measurement time (CS at T1 or a tendency to CS at T1 or F− at T2) and persistent pathological GMs or tendencies at both measurement times (CS at T1 or a tendency to CS at T1 and F− at T2).

### Ethical statement

The study was conducted in accordance with the Declaration of Helsinki and approved by the Ethics Committee of the University Duisburg-Essen (protocol code 18-8388-BO).

### Informed consent

Patient consent was not required due to the retrospective analysis of data sets on clinical routine data (Ethics Committee of the University Duisburg-Essen).

## Results

### Sample description

131 VPI met the inclusion criteria at T1 and T2. The median PMA at birth was 29 + 5 weeks and median birth weight was 1140 g (Table 1). The cohort included 51.1% males and 27.5% multiples. The median APGAR at 10 min was 9 and the median umbilical cord pH was 7.3. The following morbidities occurred during hospital stay: 31.5% sepsis, 49.6% persistent ductus arteriosus, 7.6% intraventricular haemorrhage, 1.5% periventricular leukomalacia (PVL), 20.6% retinopathy of prematurity ≥ grade 2, 6.4% bronchopulmonary dysplasia, 1.5% necrotising enterocolitis (NEC), and 3.9% focal intestinal perforation. The surviving children who did not participate in the GMA at T1 and T2 (n = 91) showed similar clinical characteristics and comorbidities compared to this cohort except for more NEC (8% vs. 1.5%, p < 0.05) and less PVL (0% vs. 4.6%, p < 0.05). The median age at assessment at T1 was 35.0 weeks of gestation and at T2 13 weeks CA. The composition of the reduced sample for T1–T3 was slightly different for the individual BSID-III scale (cognition, fine and gross motor); however, the median age at T3 was the same, at 12 corrected months. Parental education was defined as highest educational qualification held by either parent and was classified according to the International Standard Classification of Education (ISCED) into low (level 0–2), middle (level 3–4), and high (level 5–8)^34^. In our cohort 33.1% had high, 59.5% middle and 7.4% low parental education.

**Table 1:** Descriptive characteristics of the study sample (*N* = 131).

	Median [range] or n (%)
Clinical characteristics	
Child sex, male	67 (51.1)
PMA at birth (weeks)	29 + 5 [23 + 3; 31 + 6]
Birth weight (g)	1140 [410; 2120]
Height at birth (cm)	38 [25; 47]
APGAR	
5 min after birth	8 [2; 10]
10 min after birth	9 [3; 10]
Multiple births	36 (27.5)
Umbilical cord pH at birth^a^	7.3 [6.9; 7.5]
Sepsis^b^	41 (31.5)
PDA^c^	64 (49.6)
IVH ≥ grade 2	10 (7.6)
PVL	2 (1.5)
ROP ≥ grade 2^d^	18 (20.6)
BPD, moderate and severe^e^	8 (6.4)
NEC^b^	2 (1.5)
FIP^c^	5 (3.9)
Follow-up characteristics	
T1, gestational age (weeks)	35 [33; 37]
T2, corrected age (weeks)	13 [11; 15]
T3, corrected age (months)^f,h^	12 [11; 15]
Parental education^g,i^	
High	40 (33.1)
Middle	72 (59.5)
Low	9 (7.4)

Data are presented as median [range] for continuous variables and numbers (percentage) for categorical variables.

*PMA* postmenstrual age; *APGAR* score based on appearance, pulse, grimace, activity, and respiration; *PDA* persistent ductus arteriosus; *IVH* intraventricular hemorrhage; *PVL* periventricular leukomalacia; *ROP* retinopathy of prematurity; *BPD* bronchopulmonary dysplasia; *NEC* necrotizing enterocolitis; *FIP* focal intestinal perforation.

Descriptive statistics are based on ^a^*n* = 122, ^b^*n* = 130, ^c^*n* = 129, ^d^*n* = 87, ^e^*n* = 126, ^f^*n* = 95 and ^g^n = 121 and infants, otherwise *n* = 131.

^h^The age at T3 is the same regardless of the composition of the reduced sample for the individual BSID-III scale (cognition, fine and gross motor).

^i^Parental education is defined as the highest educational qualification held by either parent and was coded according to the International Standard Classification of Education (ISCED) into low (level 0–2), middle (level 3–4), and high (level 5–8)^34^.

### Developmental trajectory from preterm General Movements to Fidgety Movements (global character and tendency)

**Figure 3:**
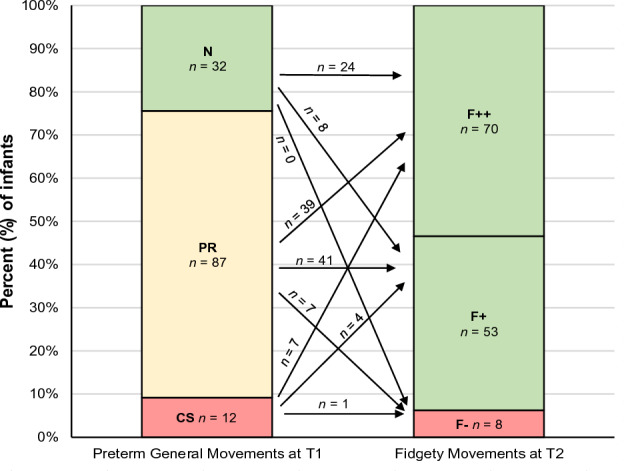
Prevalence (percentage and number of infants) and developmental trajectory of preterm General Movements global character (T1; at 35 ± 2 weeks postmentrual age) to Fidgety Movements (T2; at 12 ± 3 weeks of corrected age). N, Normal; PR, Poor Repertoire; CS, Cramped Synchronised; F++, continuous normal Fidgety Movements; F+, intermittent normal Fidgety Movements; F−, absence of Fidgety Movements. N = 131. The figures have been modified according to Prechtl and Einspieler^22,23,35^.

In the current cohort, 32 (24.4%) infants had N GMs at T1, while 87 (66.4%) had PR and 12 (9.2%) CS, as shown in Fig. 3. At T2, 123 (93.9%) infants had normal FMs, of which 70 (53.4%) had F++ and 53 (40.5%) F+, whereas 8 (6.1%) had F−. None of the infants had AFs.

Of the 32 infants with N at T1, all had normal FMs at T2, 24 (75.0%) with F++ and 8 (25.0%) with F+. Of the infants who had PR during the preterm period (n = 87), 80 (91.9%) had normal FMs (39 (44.8%) with F++, 41 (47.1%) with F+) and 7 (8.0%) had F−. Eleven (91.6%) infants with CS (n = 12) normalised at around 12 weeks CA (7 (58.3%) had F++ and 4 (33.3%) had F+), whereas 1 (8.3%) infant had F− (see Fig. 3). In summary, 112 (85.5%) infants showed no pathological GMs at both measurement times (N or PR GMs at T1 and F+ or F++ at T2). In contrast, a total of 18 infants (13.7%) exhibited abnormal GMs at one measurement time. At T1, 11 infants were categorized with CS as global character, which progressed to F+ or F++ by T2. On contrary, 7 infants showed PR at baseline and developed to F− at T2 (see arrows in Fig. 3). Finally, one infant showed persistent pathological GMs at both measurements (CS at T1 and F− at T2), accounting for 0.8% of the total sample.

Fisher-Freeman-Halton exact tests revealed a significant association between the global character of preterm GMs and FMs when considering the entire cohort of 131 infants (exact test p = 0.034) representing a small effect (Cramer’s V = 0.192). Of the infants categorised as N at T1 (n = 32), significantly more than statistically expected had continuous FMs (F++, n = 24). In addition, of the infants with PR (n = 87), significantly fewer than statistically expected had normal continuous FMs (F++, n = 39).

As can be seen in Table 2, most infants showed clear GMs global characters without tendency at T1. There was no significant association between GMs global character in combinations with tendency and FMs (p = 0.144).

**Table 2:** Prevalence and developmental trajectory of the General Movements global character in combination with tendency at T1 (35 ± 2 weeks postmenstrual age) and the Fidgety Movements at T2 (12 ± 3 weeks corrected age; N = 131).

General movements at T1			Fidgety movements at T2		
Global character	Tendency		F− *n* = 8	F+ *n* = 53	F++ *n* = 70
N	–	*n* = 32	0	8	24
N	PR	*n* = 0	0	0	0
PR	N	*n* = 25	1	11	13
PR	–	*n* = 54	5	26	23
PR	CS	*n* = 8	1	4	3
CS	PR	*n* = 1	0	0	1
CS	–	*n* = 11	1	4	6

Data are presented as numbers. *N*, normal; *PR*, Poor Repertoire; *CS*, Cramped Synchronised; F++, continuous normal Fidgety Movements; F+, intermittent normal Fidgety Movements; F−, absence of Fidgety Movements; *T1*, at age 35 ± 2 weeks; *T2*, at 12 ± 3 weeks corrected age. *N* = 131.

### Cognitive and motor BSID-III outcomes at 12 months corrected age

**Figure 4:**
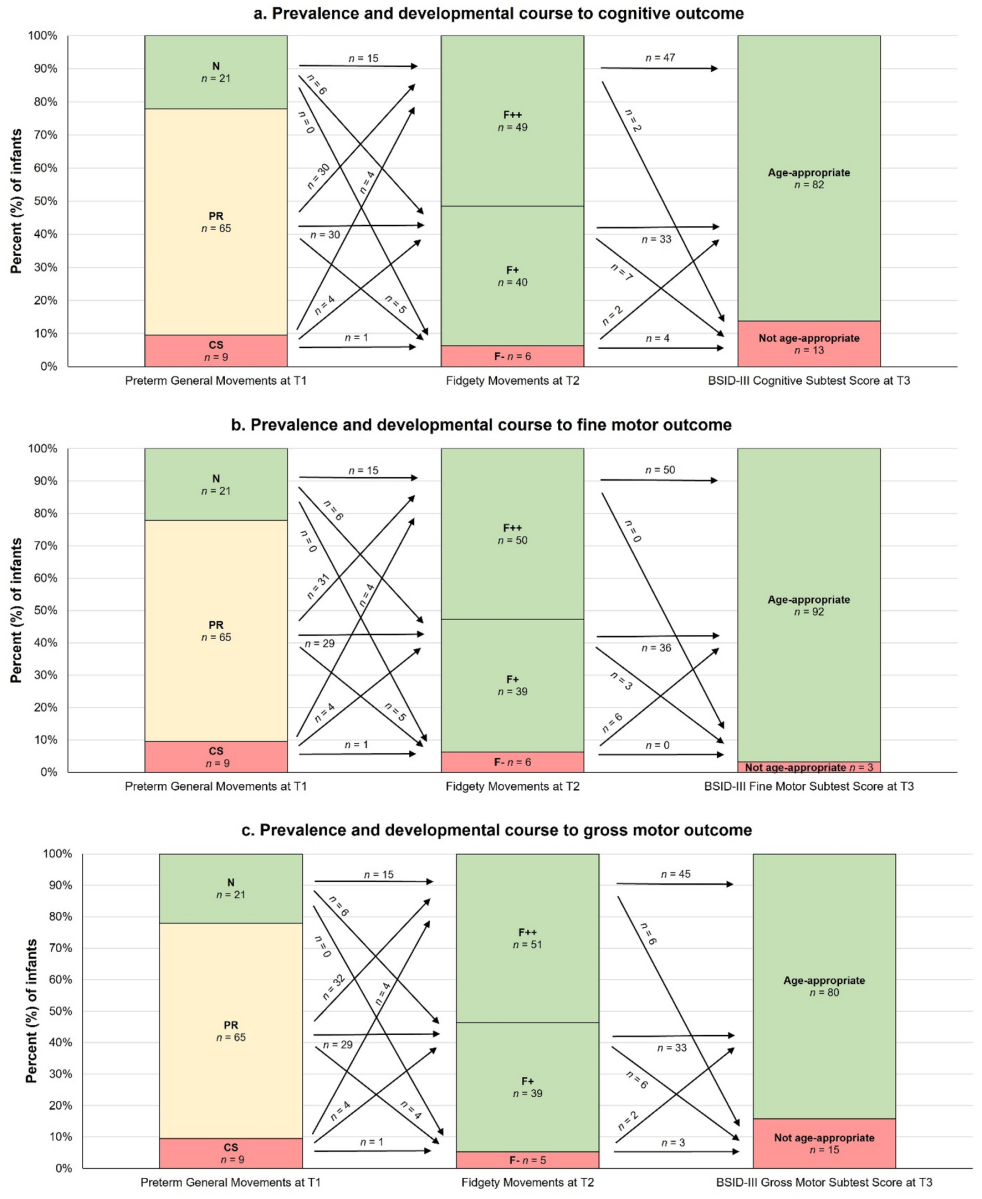
Prevalence (percentage and number of infants) and developmental course of preterm General Movements global character at 35 ± 2 weeks postmenstrual age (T1) and Fidgety Movements at 12 ± 3 weeks corrected age (T2) on a. cognitive, b. fine motor and c. gross motor BSID-III scores at 12 ± 3 months corrected age (T3). N, Normal; PR, Poor Repertoire; CS, Cramped Synchronised; F++, continuous normal Fidgety Movements; F+, intermittent normal Fidgety Movements; F−, absence of Fidgety Movements. BSID-III subtest scores between 7 and 13 were categorised as age appropriate; BSID-III subtest scores between 1 and 6 were categorised as not age-appropriate. n = 95. The figures have been modified according to Prechtl and Einspieler^22,23,35^.

The following analysis is based on reduced samples (n = 95 each), including all infants who had complete GMs at T1 and T2 and valid cognitive, fine motor and gross motor BSID-III outcome assessments. Figure 4 shows the prevalence and developmental trajectory of preterm GMs and FMs on BSID-III cognitive, fine motor and gross motor scores at T3. Fisher–Freeman–Halton exact tests revealed no significant associations between preterm GMs and FMs in these reduced samples (n = 95 each), and no significant associations between GMs global character at T1 and cognitive, fine motor and gross motor outcomes at T3.

Regarding cognitive outcomes, there was a statistical tendency for preterm GMs global characters to be associated with infants' BSID-III cognitive outcomes, representing a small effect size (exact test p = 0.064, Cramer’s V = 0.221), although this was not significant. The association became significant when considering the GMs global character together with tendency, representing a medium effect size (exact test p = 0.007, Cramer’s V = 0.366). Of those infants who had PR and no tendency (n = 40), fewer infants than expected (n = 29) had age-appropriate BSID-III cognitive subtest scores whereas more infants than statistically expected (n = 11) had non age-appropriate cognitive subtest scores.

An χ^2^ test revealed a significant association between FMs at T2 and BSID-III cognitive scores at T3 (χ^2^(2) = 18.58, p < 0.001, Cramer’s V = 0.442, medium effect). More infants than statistically expected (n = 4) did not have age-appropriate BSID-III cognitive subtest scores at T3 after scoring F− at T2 (n = 6), and fewer infants than expected (n = 2) had age-appropriate cognitive subtest scores. In addition, of those infants who showed normal continuous FMs (i.e., F++; n = 49), more than statistically expected had age-appropriate BSID-III cognitive subtest scores (n = 47) and fewer than expected (n = 2) had age-inappropriate subtest scores.

For gross motor outcomes, Fisher–Freeman–Halton exact tests revealed a significant association between FMs qualities at T2 and BSID-III subtest scores at T3 (exact test p = 0.042, Cramer’s V = 0.290, small effect). More infants than statistically expected (n = 3) did not have age-appropriate BSID-III subtest scores at T3, and fewer infants than expected (n = 2) had age-appropriate subtest scores after showing pathological F− (n = 5) at T2. In addition, of those infants who had normal FMs (n = 90), regardless of F++ or F+, more than statistically expected (n = 78) had age-appropriate BSID-III gross motor subtest scores and fewer than expected (n = 12) had age-inappropriate subtest scores. For fine motor outcomes, there were no significant associations between FMs at T2 and BSID-III subtest scores at T3 (p = 0.161).

### Tendencies and persistence of pathological GMs and cognitive and motor BSID-III scores

**Figure 5:**
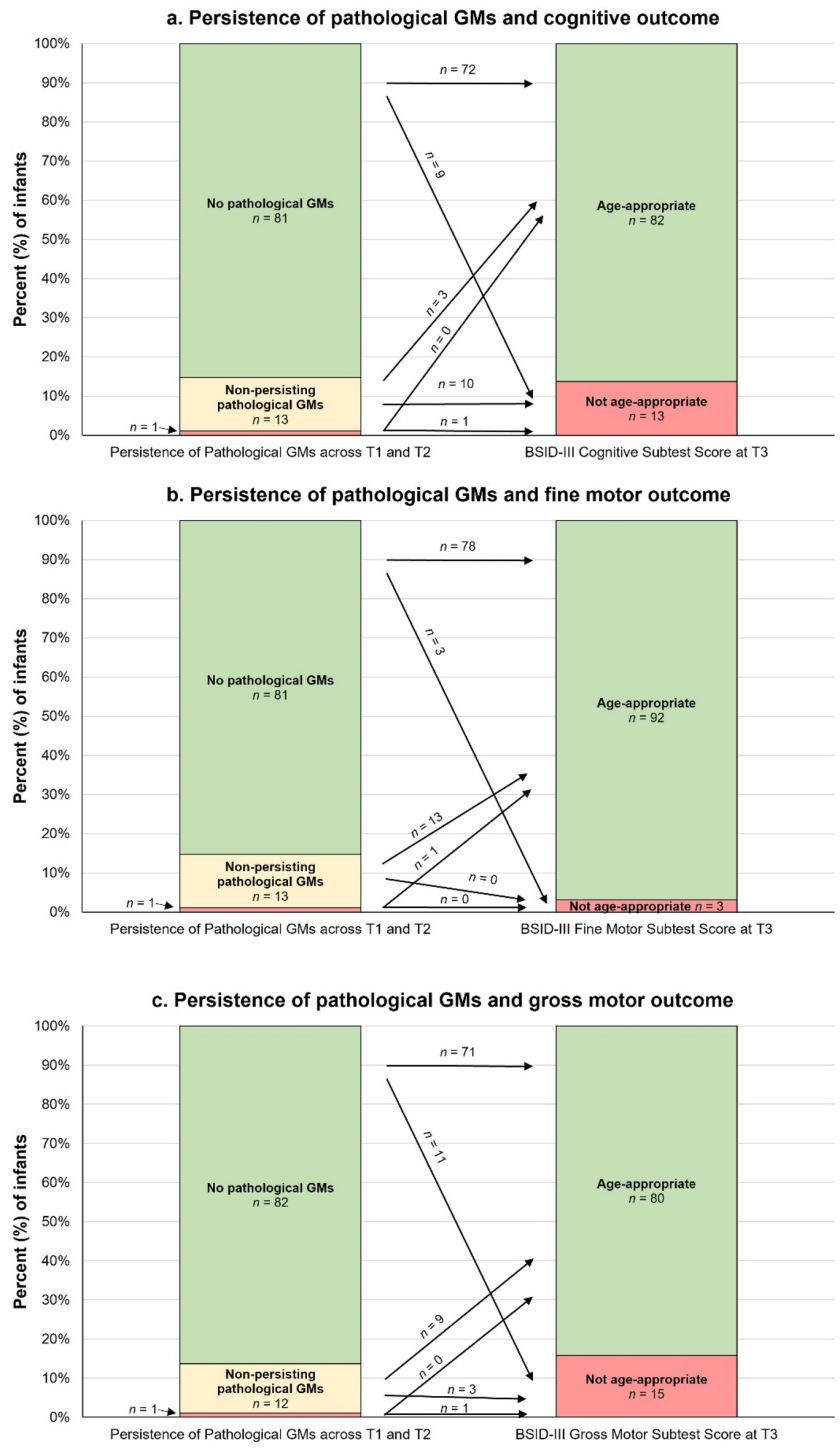
Prevalence (percentage and number of infants) and developmental course of persistence of pathological General Movements global character across T1 (at 35 ± 2 weeks postmenstrual age) and T2 (at 12 ± 3 weeks of corrected age) on (**a**) cognitive, (**b**) fine motor and (**c**) gross motor outcomes at 12 ± 3 months of corrected age (T3). The red area of the bar labelled "Persistence of pathological GMs across T1 and T2" represents the category: "persistent pathological General Movements (GMs) at both measurement times" with n = 1 for figure (**a**) cognitive, (**b**) fine motor and (**c**) gross motor outcomes, respectively. The variable persistence of pathological GMs was divided into the following categories: no pathological GMs at either assessment time (no CS at T1 and no F− at T2), no persistent pathological GMs at one assessment time (CS at T1 or F− at T2), and persistent pathological GMs at both assessment times (CS at T1 and F− at T2). BSID-III subtest scores between 7 and 13 were categorised as age-appropriate; BSID-III subtest scores between 1 and 6 were categorised as not age-appropriate. n = 95. The figures have been modified according to Prechtl and Einspieler^22,23,35^.

Figure 5 shows the persistence of pathological GMs at T1 and T2 and cognitive, fine motor and gross motor BSID-III scores at T3 in the reduced samples (n = 95 each). Although not statistically significant, there was a statistical tendency for the persistence of pathological GMs to be associated with infants' cognitive BSID-III scores, representing a small effect (exact test: p = 0.063, Cramer’s V = 0.285). This association became significant when the GMs global character (N, PR, CS) was taken into account in combination with the tendency at T1 with a medium effect size (exact test: p = 0.022, Cramer’s V = 0.368). More infants than statistically expected (n = 2) did not have age-appropriate BSID-III cognitive subtest scores whilst fewer than expected (n = 0) had age-appropriate subtest scores after showing persistent pathological GMs at both assessments (that is, CS as global character or tendency and F−; n = 2). In addition, there was a tendency for persistent pathological GMs to be associated with infants' BSID-III gross motor scores with a small effect size (exact test: p = 0.078, Cramer’s V = 0.261), although the association was not statistically significant. Including the global character of the GMs in combination with the tendency did not improve the level of significance. Finally, there were no significant associations or tendencies between the persistence of pathological GMs and fine motor outcomes at 12 months CA.

## Discussion

The developmental trajectories of GMs from preterm to fidgety age in this cohort showed at T1 predominantly monotonous less variable (PR, 66.4%) and normal (N, 24.4%) GMs followed by a higher proportion of age adequate FMs at T2 (intermitted and continuous FMs, F+ and F++, 93.9%). For cognitive outcomes, there was a statistical tendency for (a) the global character of preterm GMs (T1) and (b) the persistence of pathological GMs (T1 + T2) to be associated with infants' BSID-III cognitive scores, representing a small effect size. These associations became significant with medium-sized effects, considering the tendencies of the GMs in addition to the global character. Findings of our study indicate an association between persistent pathological GMs and cognitive outcome considering GMs tendencies in addition to global character. For gross motor skills, there was no association between preterm GMs and preterm GMs tendencies with outcome. Conversely, there were significant associations between FMs and gross motor outcomes with small effect size. Fine motor outcome showed no association between preterm GMs and FMs.

Our observations of predominant PR in the preterm period with a normalising tendency during the course is in agreement with the results of Olsen et al.^36^ who observed a higher prevalence of N GMs already at term-equivalent age. In contrast, Porro et al.^29^ report no trend towards normalisation, but rather a slight increase in pathological GMs at the term equivalent age. The prevalence of GMs global characters varies depending on the study cohorts. For instance, in 1997, Prechtl et al. refer to a significantly higher prevalence of pathology in preterm babies (44.6% PR and 30.7% CS) than more recent studies^23^. In comparison, Porro and colleagues report the dominance of N (67%) and only 3% CS^29^.

In Prechtl's cohort, there were significantly more children (46.2%) at high risk due to severe brain damage (IVH/PVL grade 2–4 on ultrasound)^23^ than in our cohort with 9.2% severe brain damage (IVH ≥ 2, PVL cluster of punctate lesions on MRI). The significantly higher prevalence of pathology in the study by Prechtl should be considered in the light of the substantial difference in perinatal management of VPI in the 1990s compared to current treatments. Surfactant administration reduces the risk of intraventricular haemorrhage and bronchopulmonary dysplasia. With the increase in non-invasive ventilation and decrease in mechanical ventilation, as well as the implementation of developmental care, there has been a paradigm shift in VPI care since the early 2000s^37,38^. Mendonca et al. identified clinical factors associated with abnormal GMs during the NICU: IVH grade I–II, prolonged duration of mechanical ventilation and presence of non-invasive ventilatory support and/or oxygen therapy^39^. Olsen et al. describe postnatal infections as risk factors in addition to IVH^36^. In this study, the proportion of both, severe brain injury and moderate/severe bronchopulmonary dysplasia was low, so that a subgroup analysis could not be performed due to lack of power.

At fidgety age, the prevalence of pathologies also varies to a large extend. Prechtl's cohort, again, shows a high prevalence of AF (12.3%) and F− (33.8%)^23^. Fewer pathologies are reported by Spittle et al. (22% F−), Kahraman et al. (16.7% F−, 6.3% AF) and Porro et al. (8.1% F−, 16.2% AF)^27,29,40^. Kadam et al. report a much lower proportion of pathologies (4.2% F− and 1.4% AF)^28^. The prevalence of severe brain lesions is 11.1% in Spittle, 13% in Porro and only 2.8% in Kadam^28,29,40^. In our cohort, 9.2% showed severe brain lesions, similar to those in the studies by both Spittle and Porro. Despite comparable proportions of severe brain lesions, our outcome at fidgety age is better (6.1% F−, no AF) than reported in Spittle et al. and Porro et al. These differences may be due to the fact that severe brain injuries in this cohort were defined by susceptibly weighted imaging, which is more sensitive to haemorrhage than cranial ultrasound and conventional MRI used in the forementioned studies. Most Infants with F− at T2 failed to achieve age-appropriate cognitive outcomes at 12 months CA and had significantly lower test scores compared to infants with F++/F+. In our cohort, 75% of infants with normal GMs at preterm age (T1) show continuous FMs (T2) and only 25% show intermittent FMs. Sokolow et al. describe that children with positional asymmetries or tone regulation disorders were more likely to show sporadic or intermittent FMs^41^. This suggests that preterm N GMs will most likely also lead to continuous FMs.

While the connection between FMs and BSID-III is well described, sequential GMs have not yet been extensively investigated. These may be of additional value as also concluded by Porro et al.^29^ because this procedure enables the earlier possibility of therapeutic intervention. Spittle et al. report on an association between pathological FMs and BSID-III cognitive outcome, at 24 months CA. In contrast, the global character of Writhing Movements (WMs) shows no correlation with cognitive outcomes^32^. This discrepancy could possibly be explained by the consideration of tendencies, unlike Spittle et al., Kadam et al. and Kahraman et al. report an association between pathological preterm GMs as well as pathological FMs and reduced cognitive outcome at 12, 18, and 24 months CA, respectively^27,28^.

Preterm GMs did not demonstrate a significant association with gross and fine motor skills, despite the inclusion of the tendency in our analysis. In the 12-month CA outcome, a tendency with a small effect size was found for the gross motor skills in the global character analysis, although not significant, but the inclusion of the tendency did not improve the significance level. This outcome may be consistent with findings from international studies of the effects of early physiotherapy on motor skills. Few international research confirms that early physiotherapy for suspected CP has a positive effect on motor development^42–44^. Early therapeutic treatment might have, as well, resulted in enhanced motor skills up to T3 in our cohort. On the other hand, there was no correlation between GMs and fine motor outcome, neither in the global analysis nor in the additional consideration of tendency (PR -> CS). It might be possible that no statistical correlations between GMs and the fine motor outcome were found, as the BSID-III measures mainly the quantity rather than the quality of fine motor abilities. Fine motor deficits may become apparent later in infancy as tasks become more complex and demanding.

In contrast to the studies mentioned above, our study provides new findings since we examined fine and gross motor dimensions separately. Therefore, the results can only be compared with other studies to a limited extent. Furthermore, a comparison with the results of Spittle et al. is difficult because the authors used the longitudinal study of a local reference group as a benchmark, which showed mean values above 100 for cognition, language and motor dimensions^32^. It is also difficult to compare our study with that of Kadam et al. because outcome at 12 months CA was assessed using the Developmental Assessment Scales for Indian Infants (DASII) rather than the BSID-III. The DASII is based on the BSID-III and is a standardised instrument for the assessment of Indian infants aged 0–30 months^28^. Our study shows a larger effect size for the association between FMs and cognition than between FMs and gross motor skills. This is consistent with the findings of Kahraman et al., who found that infants with F− have slightly more motor deficits than cognitive impairments. Spittle et al. also describe a relationship between WMs and FMs and motor outcome at 24 and 48 months CA. WMs may show higher sensitivity and FMs higher specificity^32^. To capture specific motor skills, longitudinal analyses of the participants should follow development up to school age.

A reliable early prediction prior to an MRI at term equivalent age would is warranted to identify infants at risk. Various diagnostic methods are available for early detection of neurological conditions, especially CP. However, it is noteworthy that MRI has a lower sensitivity (87%) compared to the GMA (98%). Further methods for early detection in preterm/term age are the aEEG (sensitivity 83%)^45^ or the Hammersmith Neonatal Neurological Examination (HNNE, sensitivity 71%)^46^. During fidgety age and afterwards the Hammersmith Infant Neurological Examination (HINE) reveals a sensitivity of 90% at CA of 3 months^13^. Furthermore, the HNNE and HINE depends to a great extent on the child´s cooperation and behavioural state. All mentioned methods have in common that the level of technical and/or personal effort is high. Therefore, these requirements are only met in a few centres with specific neuromonitoring expertise^47^.

A limitation of the study is the retrospective design. In our clinic, VPI with an increased risk of CP may have been filmed at closer intervals than infants with a lower risk. To ensure data consistency, narrow time windows were defined. During the SARS-CoV-2 pandemic, the parents of some VPI were afraid of infection and avoided in-person contacts. This resulted in a significant decrease in follow-up visits. An alternative video consultation using GMs home videos could not always ensure adequate quality standards. Only in-person visits could be used for the BSID-III data, as this was the only way to ensure standardised examination. Findings of our study indicate an association between persistent pathological GMs and cognitive outcome considering GMs tendencies in addition to global character. However, given that pathological movements were underrepresented in our study, more research is needed to replicate our findings in larger sample cohorts before generalizing our results and to be able to recommend sequential GMA for clinical routine.

This study provides the first evidence that taking into account the tendencies of GMs can improve the prediction of neurodevelopmental outcomes compared to global character alone. Tendencies can be easily applied in a time-neutral manner in clinical practice in contrast to scores such as the GMOS. The advantage could lie in the identification of infants at risk, which in turn opens a therapeutic window for intervention while they are still in hospital. This possible risk stratification may also offer the advantage of saving increasingly scarce human resources through individualised, needs-based therapy.

### Perspective

More intensive performance of GMA and attention to GMs tendencies could lead to better prediction of later outcome based on GMA. This might be particularly important in initially severe pathological GMs and PR GMs, as shown by Porro et al. This could be based on a prospective analysis of a larger, ideally multicentre collective. A fixed schedule with defined time points could allow (1) a standardised procedure to detect persistent pathology and (2) recognition of the timing of normal findings. GMs could be assessed fortnightly at preterm age^35^ to follow infants with PR, with CS, and with PR -> CS or CS -> PR more intensively and to enable detection of the timing of existing changes more precisely. The cost–benefit ratio should also be investigated. The use of automated analysis of visual form perception through machine learning could relieve limited resources. Initial results are already available for the fidgety age group^48^. Early identification of infants at risk for later non age-appropriate outcome could protect resources, as only infants in need would receive therapy. In addition, the effectiveness of early interventions in the first months of life requires further investigation.

## Conclusion

In this study, considering the tendency of GMs (predominantly PR interrupted with short CS sequences) seems to add value. This supplementary assessment parameter may identify infants with milder motor and/or cognitive impairments at an early stage. This research suggests a potential association between persistent pathological GMs and cognitive outcome at 12 months CA. To verify this presumption and ascertain the validity of the global character and tendencies of GMs on cognitive and motor outcomes, prospective replication in a longitudinal design with a larger cohort is needed.

## Data Availability

The dataset used and/or analysed for the study is available from the corresponding author upon reasonable request.
